# Genome-Wide Identification of the Sulfate Transporters Gene Family in Blueberry (*Vaccinium* spp.) and Its Response to Ericoid Mycorrhizal Fungi

**DOI:** 10.3390/ijms25136980

**Published:** 2024-06-26

**Authors:** Mei Dong, Jiawei He, Xiaoxuan Tang, Siwen Liu, Jinjie Xing, Xuyang Chen, Li Chen, Yadong Li, Haiyue Sun

**Affiliations:** 1 College of Horticulture, Jilin Agricultural University, Changchun 130118, China; dongmei4790@163.com (M.D.); tangl0106@163.com (X.T.); 13997481786@163.com (S.L.); xingjinjie888@163.com (J.X.); 15004917050@163.com (X.C.); chenli@jlau.edu.cn (L.C.); 2 College of Life Sciences, Jilin Agricultural University, Changchun 130118, China; 3 High Mountain Economic Plant Research Institute, Yunnan Academy of Agricultural Sciences, Lijiang 674110, China; hjw@yaas.org.cn

**Keywords:** blueberry, *Vaccinium*, sulfate transporters, genome-wide identification, mycorrhiza

## Abstract

Sulfur metabolism plays a major role in plant growth and development, environmental adaptation, and material synthesis, and the sulfate transporters are the beginning of sulfur metabolism. We identified 37 potential *VcSULTR* genes in the blueberry genome, encoding peptides with 534 to 766 amino acids. The genes were grouped into four subfamilies in an evolutionary analysis. The 37 putative VcSULTR proteins ranged in size from 60.03 to 83.87 kDa. These proteins were predicted to be hydrophobic and mostly localize to the plasma membrane. The *VcSULTR* genes were distributed on 30 chromosomes; *VcSULTR3;5b* and *VcSULTR3;5c* were the only tandemly repeated genes. The *VcSULTR* promoters contained *cis*-acting elements related to the fungal symbiosis and stress responses. The transcript levels of the *VcSULTRs* differed among blueberry organs and changed in response to ericoid mycorrhizal fungi and sulfate treatments. A subcellular localization analysis showed that VcSULTR2;1c localized to, and functioned in, the plasma membrane and chloroplast. The virus-induced gene knock-down of *VcSULTR2;1c* resulted in a significantly decreased endogenous sulfate content, and an up-regulation of genes encoding key enzymes in sulfur metabolism (*VcATPS2* and *VcSiR1*). These findings enhance our understanding of mycorrhizal-fungi-mediated sulfate transport in blueberry, and lay the foundation for further research on blueberry–mycorrhizal symbiosis.

## 1. Introduction

Blueberries (*Vaccinium*) are known for their excellent health benefits, which are related to the flavonoids in their fruits [1,2]. Highbush blueberries, lowbush blueberries (*V*. *angustifolium*), and rabbiteye blueberries (*V*. *ashei*) are the three most widely cultivated groups [3]. Highbush blueberries can be divided into southern highbush blueberries (*V*. *australe*), half-highbush blueberries (*V*. *corymbosum* × *V. angustifolium*), and northern highbush (*V. corymbosum*) blueberries, depending on their winter hardiness and chilling requirements [4]. Blueberry plants have a fibrous root system with a low absorption capacity for water and nutrients [5]. An association with mycorrhizal fungi is required to improve the absorption of mineral nutrients. Moreover, when cultivating blueberry plants, it is usually necessary to add sulfur to adjust the soil pH, because blueberry plants grow best in acidic soil [6]. The sulfur application rate differs among different regions. Hence, it would be useful to understand the absorption and metabolism of sulfate for blueberry.

Sulfur is an essential macronutrient for plant growth and development, and it plays a fundamental role in metabolism [7,8]. In addition to being a structural component of protein disulfide bonds, sulfur is present in amino acids (cysteine and methionine), vitamins (biotin and thiamin), cofactors (S-adenosyl-methionine), and the iron–sulfur groups of electron transport chains [9]. Plants use sulfur primarily in its anionic form, sulfate (SO_4_^2−^), which is generally present in very small amounts in the soil. Furthermore, because sulfate is water-soluble, it readily leaches out of the soil. Plants are able to take up sulfate from the soil over a wide range of concentrations through the use of high-affinity and low-affinity transporters [10]. In soils with low-sulfur availability, a symbiotic association between plants and arbuscular mycorrhizal fungi (AMF) assists with sulfur acquisition from the soil. Plants obtain nutrients from their fungal partner, which in return receives sugars from the plant [11,12,13]. In this process, sulfate transporters play an essential role in growth, nutrient absorption, and metabolism in plants.

Sulfate transporter (*SULTR*) genes have been characterized in many plant species, such as *Arabidopsis thaliana* [14], *Phaseolus vulgaris* [15], *Malus domestica* [16], *Camellia sinensis* [17], and *Triticum turgidum* [18]. In previous studies, the *SULTR* gene family has been divided into the following four groups according to the localization and role of the encoded protein: high-affinity SULTRs that absorb sulfate from soil, low-affinity SULTRs that transport sulfate from the roots to the aboveground plant parts, chloroplast-localized SULTRs that mainly transport sulfate within chloroplasts, and vacuole-localized SULTRs that are responsible for the outward transport of sulfate from vacuoles [19,20]. All of these transporters contain sulfate transporter anti-sigma domains in the C-terminal region [21].

In addition to studies identifying members of the *SULTR* gene family in diverse plant species, an increasing number of studies have characterized the function of *SULTRs*. Under sulfur-deficiency stress, tobacco plants (*Nicotiana tabacum*) overexpressing *GmSULTR1;2b* from soybean showed reduced yield losses [22]. Xun et al. [16] demonstrated that *MhSULTR3;1a* was specifically expressed in the root, and its encoded product was capable of transporting sulfate in a yeast functional complementation experiment. In rice, two low-affinity *SULTRs*, *OsSULTR2;1* and *OsSULTR2;2*, were shown to have sulfate transporter activity, with the activity of the former being higher than that of the latter [23]. To date, however, no study has explored the function of sulfate transporter genes in blueberry. The identification and functional validation of sulfate transporter genes in blueberry will provide mechanistic insight into how its symbiont, ericoid mycorrhizal fungi (ERMF), promotes sulfur absorption and metabolism.

In this study, we conducted a genome-wide analysis to detect genes encoding sulfate transporters in *V*. *corymbosum* and analyzed the response of *VcSULTRs* to mycorrhizal fungi and sulfates. We identified 37 sulfate transporter genes in *V. corymbosum* and evaluated their biophysical features, gene architecture, conserved motifs, distribution on the chromosomes, and transcript profiles in response to inoculation with ERMF or sulfate treatments. The results of this study provide useful information for further research on the biological functions of sulfate transporters in blueberry, especially their roles during the establishment and development of the ERMF symbiosis.

## 2. Results

### 2.1. Identification of SULTR Genes in the Blueberry Genome

A total of 37 *VcSULTR* genes were identified in the blueberry genome (Table S1). The genes were predicted to encode polypeptides of 534 (VcSULTR3;5a) to 766 (VcSULTR3;5c) amino acids, with a predicted molecular mass ranging from 60.02 to 83.97 kDa, and a theoretical isoelectric point (pI) ranging from 7.55 (VcSULTR4;1c) to 9.57 (VcSULTR2;2b). The instability index of most of the putative proteins was lower than 40, indicating that they are stable proteins. All of the VcSULTR proteins were predicted to be hydrophobic on the basis of their positive grand average hydropathicity (GRAVY) values. Details of the secondary structures of the putative proteins, including the numbers of alpha helixes, beta turns, random coils, and extended strands, are summarized in Table S1. Most VcSULTRs were predicted to localize to the cytoplasmic membrane, and a few were predicted to localize to the vacuole (Table S2).

### 2.2. Phylogenetic Analysis of VcSULTRs

**Figure 1:**
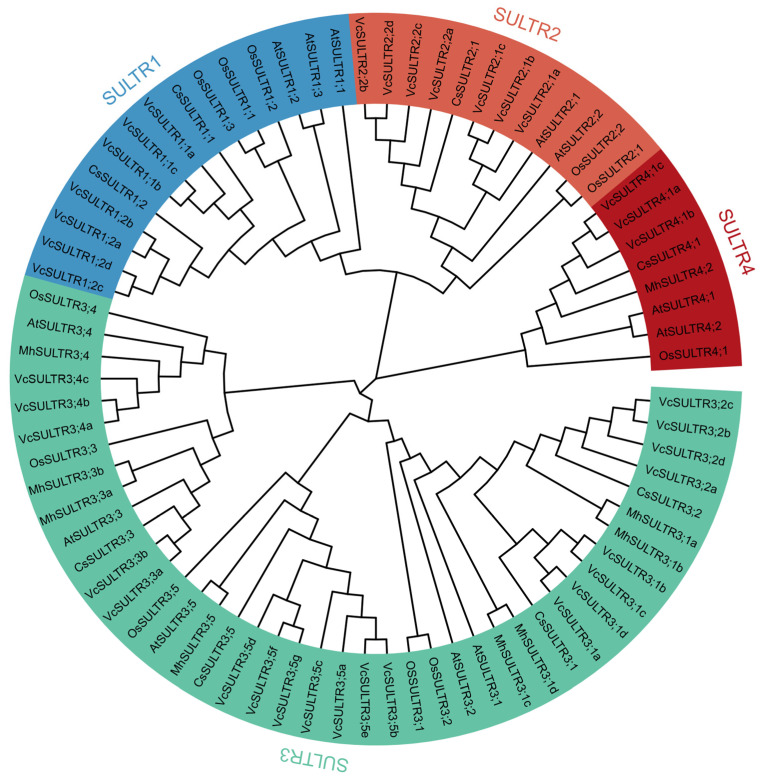
The phylogenetic tree of the SULTRs from *Vaccinium corymbosum* (Vc), *Arabidopsis thaliana* (At), *Oryza sativa* (Os), *Malus domestica* (Mh), and *Camellia sinensis* (Cs). Gene IDs are shown in Table S4.

To explore the evolutionary relationships of blueberry SULTR proteins, a multiple alignment analysis of the full-length SULTR protein sequences of *V*. *corymbosum* (37 VcSULTRs), *A*. *thaliana* (12 VcSULTRs), *M*. *domestica* (9 VcSULTRs), *C*. *sinensis* (8 VcSULTRs), and *O*. *sativa* (11 VcSULTRs) was conducted using MEGA 11. The gene IDs and corresponding gene names are listed in Table S3. The SULTR proteins in blueberry were classified into four subfamilies in the phylogenetic tree and each subfamily contained VcSULTRs (Figure 1). Group I contained VcSULTR1s that grouped with AtSULTR1s, so we hypothesized that VcSULTR1s are high-affinity sulfur transporters that absorb sulfate from the external environment. Because VcSULTR2s grouped with AtSULTR2s, they were predicted to be low-affinity sulfur transporters that transport sulfate from the roots to the aboveground plant parts. The SULTR3 subfamily contained the most VcSULTRs (20 VcSULTRs), which may localize to the chloroplasts and participate in the absorption and transport of sulfate in the chloroplast envelope. The remaining VcSULTRs clustered together with AtSULTR4s, suggesting that they localize to the vacuole membrane and promote the outward transport of sulfate from the vacuole.

### 2.3. Chromosomal Location of VcSULTRs and Collinearity Analysis

**Figure 2:**
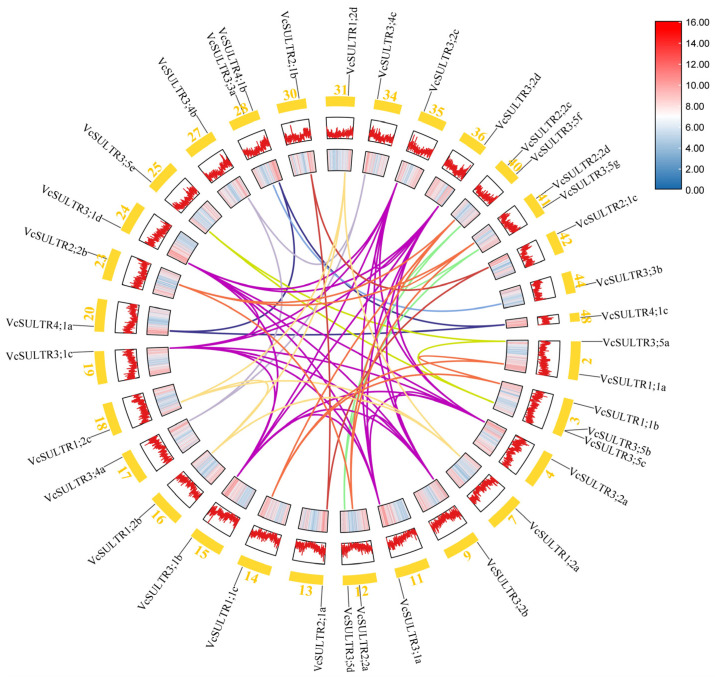
Chromosomal location and intraspecific collinearity analysis of *VcSULTRs*. The lines with different colors represent the duplicated *VcSULTR* gene pairs with collinearity relationships, and each color represents a group. The heatmap and line in the outer circle indicate gene density on the chromosome. The chromosome number is shown in the bottom of each chromosome.

**Figure 3:**
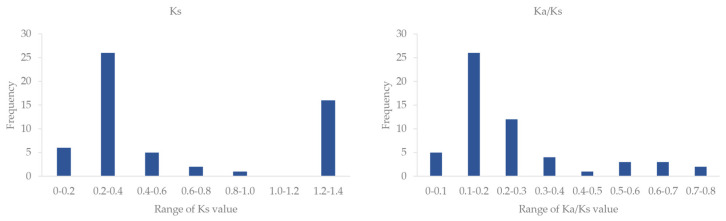
The frequency of Ks and Ka/Ks values in the *VcSULTRs*. The full details of the duplicated *SULTRs* are provided in Table S4.

The 37 *VcSULTRs* were distributed on 30 chromosomes (chr). Among them, chr3 had three *VcSULTR*s; chr2, chr28, chr40, and chr41 each had two *VcSULTRs*; and the remaining chr had one gene each. The collinearity analysis of the *VcSULTR* gene family predicted a total of 60 duplicated gene pairs. Only *VcSULTR3;5b* and *VcSULTR3;5c* were tandemly repeated genes (Figure 2), which are known to play an important role in gene family expansion. To investigate the events that have occurred within the *VcSULTR* gene family, we calculated the Ks, Ka, and Ka/Ks ratio for each duplicated gene pair. The Ks value ranged from 0.002 to 1.381, and the Ka/Ks value ranged from 0.05 to 0.76, suggesting that the genes have been subject to purifying selection during evolution. The first duplication event occurred approximately 100 million years ago (Figure 3 and Table S4).

**Figure 4:**
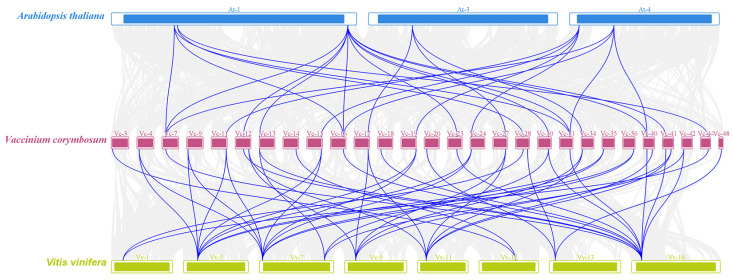
Interspecific collinearity analysis of *VcSULTRs*. Blue, purple, and green represent *Arabidopsis thaliana*, *Vaccinium corymbosum*, and *Vitis vinifera*.

In addition, an interspecific collinearity analysis was conducted with *A. thaliana* and *V. vinifera* to explore the variability and conservation of *SULTR* genes during species evolution. During the evolutionary process of the *SULTR* family gene members in blueberry, there was a higher degree of conservation between blueberry and grape, but a greater variability between blueberry and *A*. *thaliana*. The results indicated that the distribution of collinear loci was non-uniform. We detected a total of 18 collinear loci between *V. corymbosum* and *A*. *thaliana*, and 32 collinear loci between *V. corymbosum* and *V*. *vinifera* (Figure 4).

### 2.4. Conserved Motifs and Gene Structure of VcSULTRs

**Figure 5:**
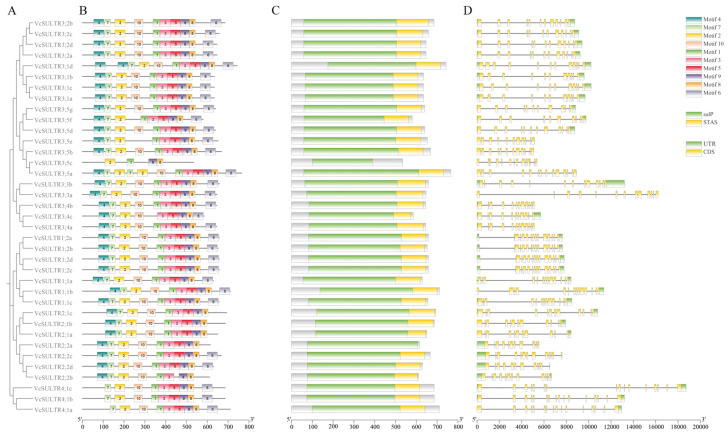
Phylogenetic relationship, conserved motifs, and gene structures analysis of *VcSULTRs*. (**A**) Phylogenetic analysis of 37 VcSULTR proteins using MEGA11. (**B**) Distributions of conserved motifs in VcSULTR proteins. Different colors represent different motifs. (**C**) The positions of Sulfate_transp (sulP) and STAS-conserved domains. (**D**) The exon–intron structure of *VcSULTR* genes. The green boxes represent untranslated regions (UTRs), the yellow boxes represent CDS (extrons), and the gray lines represent introns.

According to their genetic relationships and the position of the genes on the chromosomes, the 37 *VcSULTRs* were named *VcSULTR1;1a*–*VcSULTR4;1c*. A gene structure analysis showed that the *VcSULTRs* contained 11–16 exons. Three genes had the highest number of exons (*VcSULTR4;1a*, *VcSULTR4;1b*, and *VcSULTR4;1c*), and group III showed the largest variation in the number of exons amongst its members, indicating that members of this subfamily may have diverse functions (Figure 5). These results were consistent with the evolutionary tree results.

To further analyze differences in amino acid sequences among VcSULTRs, the conserved motifs were analyzed. Most of the VcSULTRs contained two highly conserved domains, namely the N-terminal Sulfate_transp (PF00916) domain and the C-terminal STAS (PF01740) domain. The exceptions were VcSULTR2;2a and VcSULTR3;5c, which only had the N-terminal Sulfate_transp domain. Motif analysis showed that most VcSULTRs (80%) contained motifs 1–10. VcSULTR4;1b and VcSULTR4;1c had similar motifs apart from motif 4, and VcSULTR2;2b had 8 of the 10 motifs, lacking motif 5 and motif 6. VcSULTR2;2a, VcSULTR2;1a, VcSULTR2;1b, and VcSULTR2;1c had the same motifs apart from motif 6, and VcSULTR3;5c contained only four motifs (motifs 1, 2, 8, and 9). VcSULTR3;5e and VcSULTR3;5f lacked motif 10, VcSULTR3;4c lacked motif 1, and VcSULTR4;1a lacked motif 4 (Figure 5). Thus, members of the same subfamily had similar conserved domains.

### 2.5. Identification of Cis-Regulatory Elements in VcSULTR Promoter Regions

**Figure 6:**
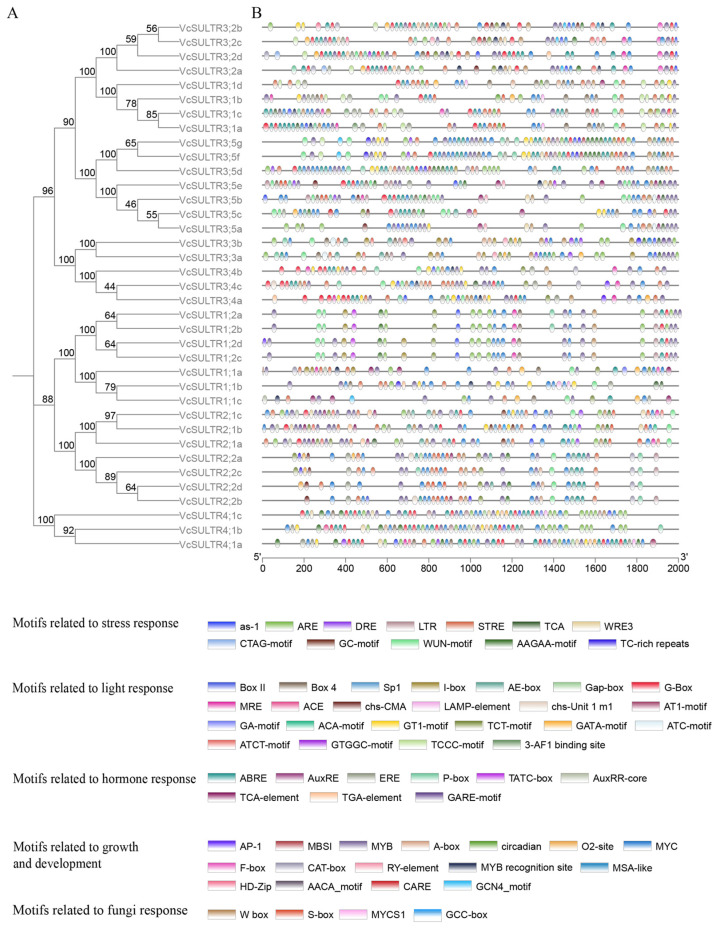
Analysis of the promoter region *cis*-regulatory element. (**A**) Phylogenetic analysis of 37 VcSULTR proteins using MEGA11. (**B**) *Cis*-regulatory elements in *VcSULTR* promoter regions.

To understand the regulation of *VcSULTR* gene expression, we identified the *cis*-acting elements within the promoter regions of blueberry *SULTR* genes (Figure 6 and Table S5). The results showed that the *cis*-acting elements from *SULTRs* could be classified into five classes according to function, including motifs related to the stress response, light response, hormone response, growth and development, and fungi response (mycorrhizal symbiosis). The *cis*-acting elements involved in the light response included G-box, GT1-box, AE box, TCCC motif, and Sp1, whereas those related to the stress response included LTR (involved in the low-temperature stress response), TC-rich repeats (involved in defense and stress responses), and MBS (involved in the drought stress response). Moreover, the promoters of *VcSULTRs* contained a large number of hormone-responsive elements, such as ABRE (involved in the abscisic acid response), ARE (induced by gibberellin), and TGA (auxin-responsive element). The *cis*-acting elements related to growth and development included MYB, MYC, and F-box. We also detected *cis*-acting elements related to the mycorrhizal symbiosis, including mycorrhizal response elements (MYCS1, S-box, and GCC box), a fungal inducer element (W box), and AuxRR core and P-box, which are involved in the auxin response.

### 2.6. Expression Patterns of SULTR Genes in Blueberry

**Figure 7:**
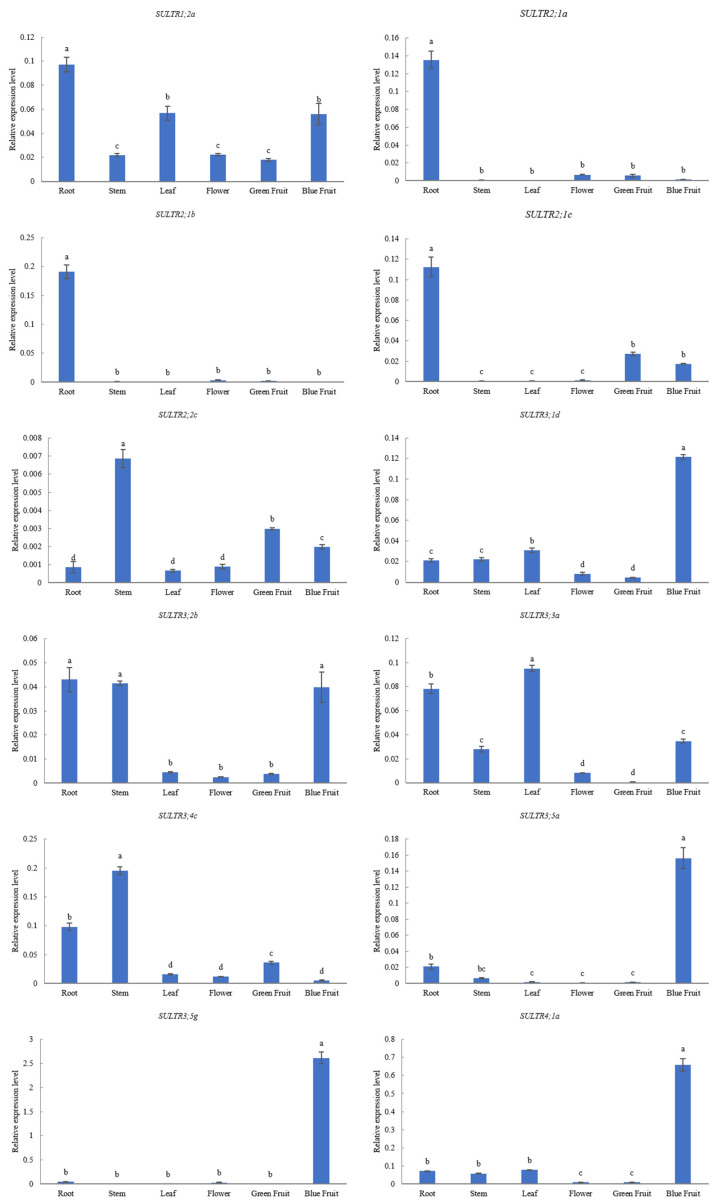
The expression levels of *VcSULTRs* in different organs of ‘Duke’ using qRT-PCR analysis. Data are presented as the mean ± SD of three independent biological replicates. Different letters indicate significant differences (*p* < 0.05).

The *SULTR* gene family plays an important role in sulfur absorption and metabolism. In the phylogenetic analysis, some sequences showed high homology. The sequences could be divided into 10 groups, so one gene from each group was randomly selected for qRT-PCR analysis. Moreover, in our preliminary experiments, we found that the homologous genes of *VcSULTR2;1a* were differentially expressed in the mycorrhizal and non-mycorrhizal roots of blueberry, so *VcSULTR2;1b* and *VcSULTR2;1c* were also used in this test. The various genes showed diverse transcript profiles in different tissues. The transcript levels of *VcSULTR1;2a* and *VcSULTR2;1a* were higher in the roots than in the other organs; those of *VcSULTR2;2c* and *VcSULTR3;4c* were higher in the stem; *VcSULTR3;3a* showed high transcript levels in the leaf; and *VcSULTR3;5a*, *VcSULTR3;5g*, and *VcSULTR4;1a* showed high transcript levels in the mature fruit (Figure 7).

### 2.7. Expression Analysis of VcSULTR Genes in Response to ERMF Inoculation and Sulfate Treatments

**Figure 8:**
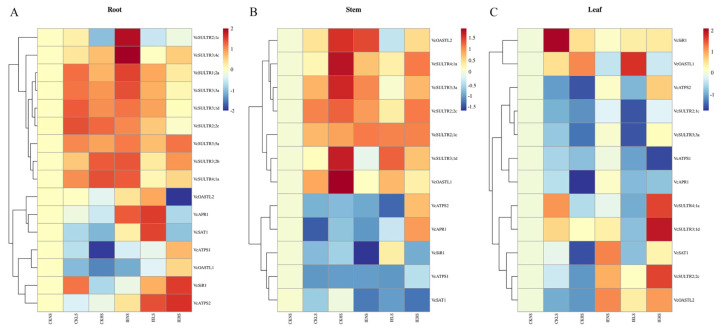
The expression levels of *VcSULTRs* and key enzyme genes for sulfur metabolism in the root, stem, and leaf under sulfate and ericoid mycorrhizal fungus treatments. CK: not inoculated; IE: inoculated with ERMF. NS: no sulfate application; LS: low-sulfate (0.01 mmol/L MgSO_4_) treatment; HS: high-sulfate (1 mmol/L MgSO_4_) treatment. (**A**) The expression levels of *VcSULTRs* and key enzyme genes for sulfur metabolism in the root. (**B**) The expression levels of *VcSULTRs* and key enzyme genes for sulfur metabolism in the stem. (**C**) The expression levels of *VcSULTRs* and key enzyme genes for sulfur metabolism in the leaf.

To compare the expression of the various genes under different sulfate levels and in response to ERMF inoculation, we quantified the transcript levels of *VcSULTRs* by qRT-PCR. The results showed that the vast majority of *SULTR* genes validated in this experiment were up-regulated in the root and stem upon sulfate treatments and ERMF inoculation, except for *VcSULTR2;1a* under CKHS treatment and *VcSULTR2;2c* under IEHS treatment in the root, and *VcSULTR3;1d* under IENS treatment (Figure 8). In the leaf, the expression of *VcSULTRs* was affected by ERMF and sulfate supply levels and exhibited diversity (Figure 8). *VcSULTR3;1d* was up-regulated upon sulfate treatments, *VcSULTR2;2c* was up-regulated by ERMF inoculation, and the other *VcSULTR* genes were both up-regulated and down-regulated in the leaf. These results indicated that *VcSULTRs* may regulate sulfur absorption at the transcriptional level and respond to mycorrhizal symbiosis, especially for *VcSULTR2;1c*, which was up-regulated under ERMF treatment.

To further explore the mechanism of sulfate absorption and metabolism, we analyzed the transcript profiles of genes encoding key enzymes in various metabolic pathways (Figure 8). Compared with uninoculated plants, those inoculated with ERMF showed increased transcript levels of *VcATPS2*, *VcAPR1*, and *VcSAT1* in the root and leaf, and decreased transcript levels of *VcATPS1* and *VcOASTL1* in those two organs. *VcOASTL2* was up-regulated in the root, stem, and leaf. *VcSiR1* was down-regulated in the root and stem, but up-regulated in the leaf.

### 2.8. Subcellular Localization of VcSULTR2;1c

**Figure 9:**
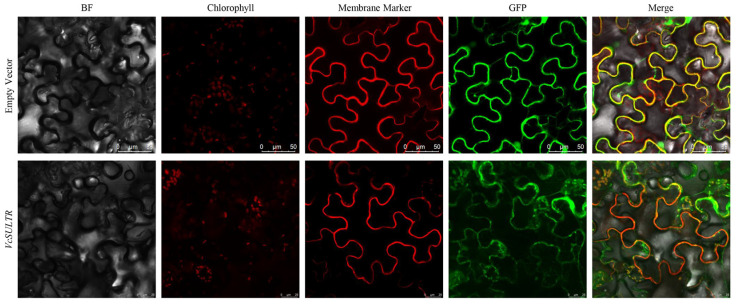
Subcellular localization of the VcSULTR2;1c protein.

Considering that *VcSULTR2;1c* was especially expressed in the roots (Figure 7) and induced by ERMF inoculation (Figure 8), and that previous studies have shown that the gene is most significantly up-regulated after inoculation with ericoid mycorrhizal fungi, *VcSULTR2;1c* was isolated from the roots of blueberry (Figure S1). To determine the subcellular location of VcSULTR2;1c, we constructed a vector expressing the GFP-VcSULTR2;1c fusion protein, which was infiltrated into tobacco leaves via *Agrobacterim tumefaciens* strain GV3101 (pSoup-p19). The GFP signals of VcSULTR2;1c were detected in the plasma membrane and chloroplast (Figure 9).

### 2.9. Expression Analysis of VcSULTR2;1c and Its Involvement in Sulfate Absorption

**Figure 10:**
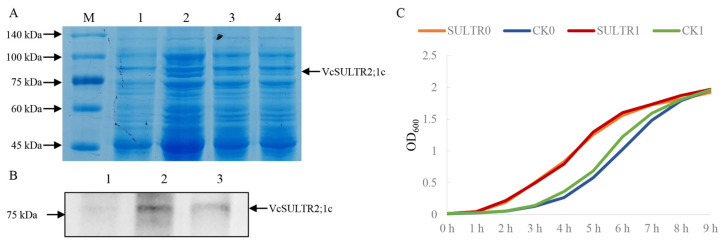
The expression of *VcSULTR2;1c* in *E. coil*. (**A**) SDS-PAGE analysis of the prokaryotic expression protein of *VcSULTR2;1c* gene. Line 1 represents the whole protein of *E. coli pEASY*-Blunt E2; lines 2–4 represent pEASY-VcSULTR2;1c recombinant proteins induced by 0.1 mmol/L, 0.2 mmol/L, and 0.4 mol/L IPTG, respectively. The arrow indicates the band of VcSULTR2;1c protein. (**B**) Western blot analysis using His-tag mouse monoclonal antibody and HRP-conjugated affinipure goat anti-mouse. 1: *E. coil pEASY*-Blunt E2 vector, 2: *E. coil* pEASY-VcSULTR2;1c whole cell, 3: *E. coil* pEASY-VcSULTR2;1c supernatant. Three samples were induced by 0.1 mmol/L of IPTG. (**C**) The growth curves of recombinant bacteria and non-recombinant bacteria. 0 represents no additional sulfur added. 1 represents adding 0.1 mol/L of MgSO_4_. CK represents the control vector. SULTR represents pEASY-VcSULTR2;1c. All samples are induced by 0.1 mmol/L of IPTG.

Analyses of gene transcript levels indicated that *VcSULTR2;1c* was significantly up-regulated after sulfate application (Figure 8), suggesting that this gene may encode a sulfate transporter. To further analyze the function of *VcSULTR2;1c*, we successfully constructed a fusion gene *pEASY-VcSULTR2;1c* (Figure S2) and performed a prokaryotic expression analysis. Western blot and SDS-PAGE analyses showed that, compared with the *Escherichia coli* strain harboring the empty vector *pEASY*-Blunt E2, the strain harboring *VcSULTR2;1c* produced a protein of ~75 kDa at 8 h after IPTG induction. The size of this band was consistent with the size of the putative VcSULTR2;1c protein (Figure 10A, B), indicating the correct expression of the recombinant protein and being a soluble protein. Growth kinetics analyses indicated that the effect of low sulfate (0.1 mol/L MgSO_4_) on the growth of *E. coli* is not significant; moreover, whether sulfate is applied or not, the *VcSULTR2;1c* significantly promoted the growth of *E*. *coli* in the short term (Figure 10C).

### 2.10. Virus-Induced Gene Silencing of VcSULTR2;1c

**Figure 11:**
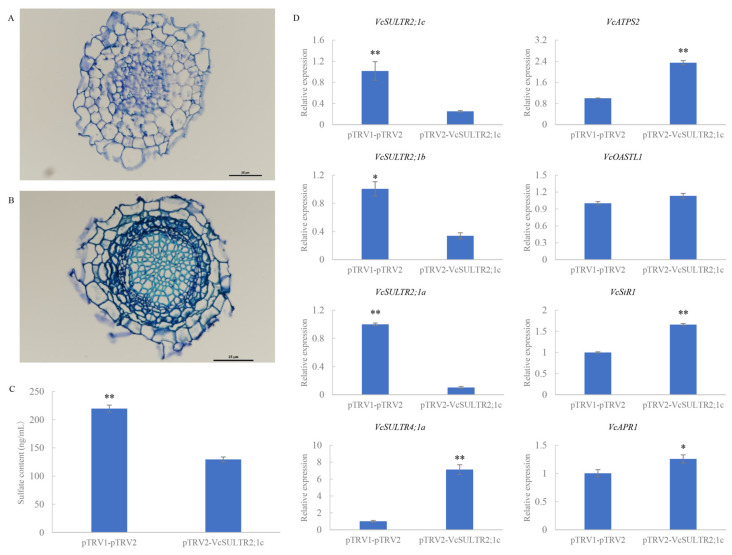
Functional analysis of *VcSULTR2;1c* in blueberry root. (**A**) Transverse section of blueberry roots with pTRV1-pTRV2. (**B**) Transverse section of blueberry roots with *pTRV2-VcSULTR2;1c*. (**C**) The effect of silenced *VcSULTR2;1c* gene on endogenous sulfate content. ** means significant difference at 0.01 level. (**D**) The expression analysis of blueberry roots silenced for *VcSULTR2;1c* gene through VIGS system. * means significant difference at 0.05 level, ** means significant difference at 0.01 level.

To explore the function of *VcSULTR2;1c* in sulfate transport, we constructed a recombinant gene *pTRV2-VcSULTR2;1c* (Figure S3) and knocked down the expression of *VcSULTR2;1c* in blueberry roots by virus-induced gene silencing (VIGS). The blueberry plants with knocked-down *VcSULTR2;1c* grew normally under the conditions of the two treatments and grew new white fibrous roots. Toluidine blue staining revealed that the root cells of the control group were arranged in a regular pattern, while the cortical cells of the experimental group were narrower, and some had a concave shape (Figure 11). qRT-PCR analyses confirmed that *VcSULTR2;1c* was strongly down-regulated in the silenced blueberry roots (Figure 11), and that the other two homologs of *VcSULTR2;1c*, namely *VcSULTR2;1a* and *VcSULTR2;1b*, were down-regulated as well. In addition, vacuole *SULTRs*, like *VcSULTR4;1a*, were up-regulated. In contrast, *VcATPS2*, *VcAPR1*, and *VcSiR1* that encode key enzymes in the sulfur metabolism pathway were significantly up-regulated. The endogenous sulfate content in the roots was significantly lower in the *VcSULTR2;1c*-knocked-down line than in the control.

## 3. Discussion

SULTR is a carrier protein for higher plants to absorb and transport sulfates. It participates in the absorption of sulfates from soil by plant roots, and it is a key factor in the transportation and redistribution of sulfates within the plant body [21]. Genome-wide analysis of the *SULTR* gene family has been performed in several species, including *M*. *domestica* [16], *C*. *sinensis* [17], *T*. *turgidum* [18], *O. sativa* [19], and *C*. *sativa* and *Brassica napus* [24]. However, no other studies have conducted a similar analysis of *VcSULTR* genes in blueberry. In this study, we identified 37 *VcSULTRs* genes in the genome of the tetraploid blueberry cultivar ‘Draper’. Our results show that, compared with the plant species mentioned above, blueberry has a higher number of *SULTR* genes. This is likely related to the polyploidy, genome size, and evolutionary history of blueberry. Analyses of the physical and chemical properties of the putative VcSULTRs revealed positive total average hydrophilicity values, indicating that all of these proteins are hydrophobic, like the SULTRs of oilseed crops and wheat [18,24]. This suggests that VcSULTRs function under the same conditions. A gene structure analysis revealed high conservation of the structure of *VcULTRs*. The motifs of most proteins were roughly the same, except for VcSULTR3;5c, which contained only motif 4. Like the SULTR proteins of most plants, VcSULTRs contained sequences encoding Sulfate_transp and/or STAS domains, which are typical characteristics of *VcSULTRs.* In addition, our analyses revealed 11–16 exons in blueberry *VcSULTRs*, different from the 4 to 20 exons in the *SULTRs* of *C*. *sativa* and 4 to 19 exons in the *SULTRs* of *B*. *napus* [24], but similar to the 11–17 exons in the *SULTRs* of *C*. *sinensis*. These results suggest that the genetic structure of blueberry may be more similar to that of *C*. *sinensis* [17].

Previous studies have divided *SULTRs* into four subfamilies, with group I and group II expressed in the roots, group III mainly expressed in the leaves, and group IV primarily expressed in vacuolar cells [16,17,24,25]. In the phylogenetic analysis, the 37 VcSULTRs were divided into four subfamilies, like the SULTRs in other species such as tea tree [17], potato [26], and sorghum [27]. The VcSULTRs were found to be distributed in all four subfamilies, although their evolutionary trends were different. According to the Ka/Ks indices, the first duplication event of *VcSULTRs* in blueberry occurred approximately 100 million years ago, which is earlier than duplication events in *C*. *sativa* and *B. napus* [24]. Furthermore, most of the *VcSULTRs* appeared to originate from *VcSULTR3*, consistent with the findings of Heidari et al. [24]. The Ka/Ks value of all the *VcSULTR* genes was less than 1, indicating that these genes may have been subject to certain limitations to maintain their function or structure, as reported for the *SULTRs* of *B. napus* [24]. Other studies have reported that there is a relatively high gene homology within each subfamily of *SULTRs*, and there is functional similarity among the proteins in each subfamily. For example, in tea, *CsSULTR1;1*. *CsSULTR1;2*, and *CsSULTR3;2* were found to be significantly up-regulated in response to an exogenous sulfur treatment. Similarly, in maize roots, sulfur deficiency significantly up-regulated *ZmSULTR1;1*. *ZmSULTR1;2*, and *ZmSULTR3;4* [17,28]. Other studies showed that *OsSULTR2;1* and *OsSULTR2;2* in rice have sulfate transport activity [23], and *PtSULTR1;1a* and *PtSULTR3;3a* in *Populus* transport sulfates in the phloem [29]. However, the proteins in the third subfamily are more diverse. We found that the third subfamily had the largest number of proteins, and they could be further classified into three groups. Chen et al. [25] found that AtSULTR3s are localized in chloroplasts, where they are involved in the uptake of sulfate and cysteine. Xun et al. [16] showed that MhSULTR3;1a localizes in plasma membranes and nuclear membranes, and suggested that its sulfur transport function can improve plant tolerance to low-sulfur conditions. In our analyses, most VcSULTRs were predicted to localize in the plasma membrane and vacuole, but a subcellular localization analysis revealed that VcSULTR2;1c localizes in the plasma membrane and chloroplasts. The above results indicate that VcSULTRs in blueberry are similar to those in other species, in that they show functional diversity and functional redundancy [16,25].

In production and cultivation, applying sulfur powder to reduce soil pH is an important part of soil improvement, which directly affects yield and economic benefits. If the application of microbial fertilizers can reduce soil pH, it can avoid a series of problems such as heavy metal pollution caused by improper sulfur application, which is beneficial for production and green environmental protection. Therefore, analyzing the response of sulfur transporter genes to mycorrhizal fungi and sulfate is of great significance. In this study, we found that the expression of *VcSULTRs* was tissue-specific and responsive to sulfate and ERMF inoculation. For instance, there were very high transcript levels of *VcSULTR2;1a/b/c* in blueberry roots; those of *VcSULTR3;2b*, *VcSULTR2;2c*, and *VcSULTR3;4c* were significantly higher in the stems than in the other organs; and *VcSULTR3;5a*, *VcSULTR3;5g*, and *VcSULTR4;1a* were specifically expressed in blue (ripe) fruits. A tissue-specific expression of *SULTRs* has also been detected in *C. sinensis* [17], *Z. may* [28], *M. domestica* [16], *C. sativa*, and *B. napus* [24], with differences in gene expression patterns among these species, and between blueberry and these species. Interestingly, we found that *VcSULTRs* in the roots were significantly up-regulated upon inoculation with ERMF and in the sulfate treatments, and this may be related to the control of gene expression via *cis*-acting elements related to mycorrhizal symbiotic and stress responses in the gene promoter regions (Figure 6), such as TC rich repeats, which is involved in the defense and stress response. In addition, we found that the elements involved in the stress response include LTR and MBS, which may be due to the intermediate products of sulfur metabolism, cysteine, and glutathione, and play important roles in resisting biotic and abiotic stress. In *Medicago truncatula*, the expression of some *MtSULTRs* was found to be induced by sulfate and mycorrhiza, which shed light on the role of mycorrhizal interactions in sulfate absorption [30]. Therefore, we speculate that *VcSULTR2s (VcSULTR2;1a*, *VcSULTR2;1b*, and *VcSULTR2;1c*) may be involved in the mycorrhizal symbiosis and have sulfate absorption and transport functions.

In recent years, the RNA-mediated plant antiviral mechanism VIGS has been widely used in reverse genetics research to investigate the function of plant genes. For example, gene knock-out or knock-down using VIGs has been used to determine the function of genes related to physiological pathways, disease resistance, growth and development, and metabolic regulation. This technology has been successfully established for fruit trees, for example, pear [31], apple [32], grape [33], and blueberry [34]. In this study, we aimed to silence gene expression in blueberry roots, which differ from fruits and leaves, so we selected the vacuum infiltration method. The knocked-down expression of *VcSULTR2;1c* in the roots resulted in gaps between epidermal cells, a wrinkled and deformed phenotype of cells in the endothelial layer, and decreases in the endogenous sulfate content and the transcript levels of *VcSULTR2;1c*, *VcAULTR2;1b*, and *VcSULTR2;1a*. However, *VcATPS2*, *VcAPR1*, and *VcSiR1* were up-regulated in the roots; moreover, *VcSULTR4;1a*, the vacuole *SULTR*, was up-regulated. These results indicate that the knock-down of *VcSULTR2;1c* either inhibited the transport of sulfate from the roots to the aboveground parts, or promoted sulfate metabolism in the roots, and may transport sulfates to the vacuoles for storage. When *VcSULTR2;1c* was expressed in *E. coli*, a 75 kDa protein consistent with the size of VcSULTR2;1c was produced, and it belongs to soluble proteins. Furthermore, the recombinant bacteria grew better than the empty control with and without sulfate, as demonstrated by the growth kinetics analysis. Sulfur is one of the essential trace elements for the growth of *E. coli* and is crucial for maintaining individual growth. Research has shown that sulfate transporters are responsible for the absorption of sulfates and thiosulfates, providing a sulfur source for sulfur metabolism, thereby synthesizing substances such as sulfur-containing amino acids and coenzymes that are crucial for growth and development [7,21,35]. This study found that recombinant bacteria significantly promoted the growth of *E. coli*. Therefore, we speculate that the *VcSULTR2;1c* may promote sulfur metabolism in *E. coli*, generating beneficial growth and development compounds such as cysteine and sulfides, thereby promoting the growth of *E. coli* in the early stages. Further research, including the use of methods such as yeast complementation analysis and homologous transformation [16,23,36], are needed to further explore the role of *VcSULTR2;1c* in sulfate transport.

## 4. Materials and Methods

### 4.1. Plant Materials and Treatments

For tissue-specific gene expression analyses, samples of different organs were collected from 6-year-old plants of the blueberry cultivar ‘Duke’ growing at the Blueberry Germplasm Resource Nursery of Jilin Agricultural University in Changchun, China (43°79′ N, 125°41′ E). All samples were immediately frozen in liquid nitrogen and then stored at −80 °C.

Plants were grown from cuttings for analyses of gene expression in response to ERMF (*Oidiodendron maius*) inoculation and sulfate treatments. Two-year-old plants with consistent growth were selected for use in these experiments, which were conducted in a greenhouse. There were three sulfate treatments, no sulfate (0 mmol/L MgSO_4_), low sulfate (0.01 mmol/L MgSO_4_), and high sulfate (1 mmol/L MgSO_4_), represented by NS, LS, and HS, respectively. In addition, the plants were inoculated or not inoculated with ERMF under these three sulfate treatments, making a total of six treatments. Each treatment was repeated three times with 10 seedlings per replicate. The substrate had sterile treatment to eliminate interference from soil fungi. The inoculum of ERMF was cultured in 75 mL of malt extract broth (MEB) for 10 days until the mycelium occupied about two-thirds of the volume of the MEB, and then they were made into a homogenate with a homogenizer and was applied as an inoculum [37]. For the first inoculation, 25 mL of homogenate was added 5 cm apart from blueberry roots. After one month, we conducted the second inoculation like the first time. The irrigation of sulfate solutions and inoculation were carried out simultaneously. To promote mycorrhizal colonization, we applied 20 μmol/L of KH_2_PO_4_ once a week [38]. All other conditions were those used in conventional field management. We collected the roots, stem, and leaf samples three months after the second inoculation. All samples were quickly frozen in liquid nitrogen and stored at −80 °C.

### 4.2. Identification of SULTR Gene Family Members in Blueberry

Two methods were used to identify the *SULTR* family members in blueberry. First, the genomic data for blueberry were obtained from the *Vaccinium* database (https://www.vaccinium.org/analysis/49, accessed on 18 March 2023) [39]. The protein sequences of the *A. thaliana SULTR* gene family were downloaded from TAIR (https://www.arabidopsis.org/, accessed on 18 March 2023) and used as BLASTP templates (E-value of 1 × 10^−5^) to identify all *SULTR* candidate members in the blueberry genome. Second, the Hidden Markov Model (HMM) files of the SULTR structural domains (PF00916 and PF01740) were downloaded from the Pfam database (https://www.ebi.ac.uk/interpro/download/Pfam/, accessed on 18 March 2023). We searched for *SULTR* genes in the *V. corymbosum* genome database using the Hmmer search function in the HMMER 3.0 program (default parameters) [40,41,42]. The results of the two methods were compared, and those genes identified using both methods were retained. Finally, the SULTR-conserved structural domains of all candidates were retrieved using the online tool NCBI CD-search (https://www.ncbi.nlm.nih.gov/Structure/bwrpsb/bwrpsb.cgi, accessed on 18 March 2023). Proteins with incomplete SULTR structural domains were excluded, and the remaining SULTR members were designated as *VcSULTRs*.

### 4.3. Gene Information and Phylogenetic Relationships

The amino acid sequence length, molecular weight (MW), theoretical isoelectric point (pI), and grand average of hydropathicity index (GRAVY) of VcSULTRs were analyzed using the ExPASy Prot-Param tool (https://web.expasy.org/protparam/, accessed on 18 March 2023) [43]. Protein secondary structures were predicted by SOPMA (https://npsa-prabi.ibcp.fr/cgi-bin/npsa_automat.pl?page=npsa_sopma.html, accessed on 18 March 2023) [44]. The subcellular localization of VcSULTRs was predicted using WoLF PSORT (https://www.genscript.com/wolf-psort.html, accessed on 18 March 2023) [45]. The ClustalW tool was used to perform multiple sequence alignments of blueberry VcSULTRs and SULTR proteins from *A. thaliana*, *M*. *domestica*, *C*. *sinensis*, and *O*. *sativa*. A phylogenetic tree was constructed using the neighbor-joining (NJ) method in MEGA 11 software with a bootstrap value of 1000 [46].

### 4.4. Chromosomal Location of Genes and Collinearity Analysis

GFF3 files and genome sequence files were used for these analyses. The chromosomal locations of *SULTR* family members were visualized using the Gene Location Visualize From GTF/GFF tool in TBtools software (v2.062). The candidate genes were renamed from *VcSULTR1;1a* to *VcSULTR4;1c* according to the phylogenetic results and their positions on the chromosomes [47]. The collinearity of *SULTR* family members was visualized using the Advanced Circos tool and Multiple Synteny Plot tool in TBtools software (v2.062). The collinearity of the *SULTR* gene family between *V*. *corymbosum*, *A*. *thaliana*, and *V*. *vinifera* was visualized using the Multiple Synteny Plot tool in TBtools software (v1.112).

### 4.5. Analysis of Domain Motifs and Gene Structures

The online program MEME Suite 5.5.1 (https://meme-suite.org/meme/tools/meme, accessed on 18 March 2023) was used to analyze conserved motif structures. The number of motifs was 10, and the width of motifs was set to the default value. Then, NCBI-CD (https://www.ncbi.nlm.nih.gov/Structure/bwrpsb/bwrpsb.cgi, accessed on 18 March 2023) was used to confirm the presence of conserved domains. The conserved motifs and domains in candidate genes were visualized using TBtools software (v1.112) [47].

### 4.6. Analysis of VcSULTR Gene Promoter Regions

The *cis*-acting elements in the promoter fragments of the *VcSULTR* genes (2000 bp upstream of the translation initiation sites) were identified using the online program PlantCARE (http://bioinformatics.psb.ugent.be/webtools/plantcare/html/, accessed on 20 March 2023). Several other *cis*-acting elements were identified according to previous studies [48,49,50]. The results of these analyses were displayed using TBtools software (v1.112) [47].

### 4.7. Expression Pattern Analysis of VcSULTRs

To understand the expression patterns of the identified *VcSULTR*, we determined the transcript levels of *VcSULTRs* in different organs of blueberry plants and under different conditions, including low sulfate (0.01 mmol/L MgSO_4_) and high sulfate (1 mmol/L MgSO_4_), and not inoculated or inoculated with ERMF (*Oidiodendron maius*) [51]. qRT-PCR was performed with TBGreen with the ABI StepOne Plus system. The *EF1α* gene was used as the housekeeping gene. All primers used in this study are listed in Table S6. The tissue-specific gene expression analyses were calculated using the 2^−ΔCt^ method. For the response to sulfate and ERMF, the relative transcript level of each gene was calculated using the 2^−ΔΔCt^ method [52,53].

### 4.8. Cloning of VcSULTR2;1c and Subcellular Localization

First-strand cDNA was synthesized from the total RNA extracted from blueberry root samples. The complete coding sequence of the *VcSULTR2;1c* gene was cloned using the primers shown in Table S6. For subcellular localization analysis of VcSULTR2;1c, the plasmid pGDG-VcSULTR2;1c and empty vector were each introduced into *Agrobacterium tumefaciens* GV3101(pSoup-p19) by the liquid nitrogen quick-freezing method, and then transfected into tobacco leaves. The injected tobacco plants were cultured under a low light level for 48 h and then fluorescence signals were detected by fluorescence confocal microscopy. The two vectors were transformed into tobacco leaves with a cytoplasmic membrane marker [54].

### 4.9. Prokaryotic Expression of VcSULTR2;1c

The *VcSULTR2;1c* fragment was amplified and ligated into the *pEASY*-Blunt E2 vector, which was subsequently transformed into competent *Escherichia coli* Rosetta. The positive recombinant transformant was cultured in Luria–Bertani broth containing 50 μg/mL of kanamycin with shaking at 37 °C until OD_600_ = 0.6–0.8, and then induced with IPTG. The total cellular pellet, pellet, and supernatant were analyzed by 10% SDS-PAGE. After electrophoresis, one gel was stained with Coomassie Brilliant Blue, and the other was used for Western blot analysis with a His-tag mouse monoclonal antibody and HRP-conjugated affinipure goat anti-mouse IgG (H + L). After IPTG induction, the bacterial solution was diluted 100 times and inoculated into LB broth containing different concentrations of MgSO_4_ to observe the growth of recombinant bacteria [55].

### 4.10. Transient Transformation of Blueberry Root

For the construction of blueberry *pTRV2-VcSULTR2;1c*, the 398 bp fragment of *VcSULTR2;1c* was amplified by PCR from blueberry root cDNA as the template. The PCR product was fused to the pTRV2 plasmid. The *VcSULTR2;1c* recombinant plasmid was transformed into *A*. *tumefaciens* strain GV3101 (pSoup-p19) and then transformed into blueberry roots using the vacuum infiltration method [56]. After 15 days, the roots were collected to detect the transcript levels of *VcSULTR2;1c* and other genes in the transformed plants and the endogenous sulfate content.

## 5. Conclusions

In this study, 37 *VcSULTR* genes were identified according to blueberry genome data of ‘Draper’ and classified into four subfamilies based on phylogenetic analysis. In addition, combining phylogenetic analysis and gene structure analysis, the VcSULTR3 subfamily was the most diverse sulfate transporter family. The results of collinearity analysis show that the *VcSULTR* family was extended via segmental duplication events. The expression of blueberry *VcSULTRs* detected in this study was tissue-specific and induced by a mycorrhizal fungus and by sulfate. Subcellular localization analysis showed that VcSULTR2;1c localized in the plasma membrane and chloroplast, and *VcSULTR2;1c* might be involved in sulfate transport. These findings provide new insights into sulfate transporters in blueberry. However, further functional studies are needed to reveal the roles of *VcSULTRs* in sulfate transport and in mycorrhizal symbiosis.

## Data Availability

The original contributions presented in the study are included in the article/Supplementary Material, further inquiries can be directed to the corresponding authors.

## Supplementary Materials

The following supporting information can be downloaded at: https://www.mdpi.com/article/10.3390/ijms25136980/s1.
