# Supplementary material for: Genome-Wide Identification of the Sulfate Transporters Gene Family in Blueberry (Vaccinium spp.) and Its Response to Ericoid Mycorrhizal Fungi
Source: Int J Mol Sci. 2024 Jun 26;25(13):6980. doi: 10.3390/ijms25136980 (PMC11241426; doi:10.3390/ijms25136980)
Supplement: Supplementary file 1 [file ijms-25-06980-s001.zip › Figure S1.pdf]

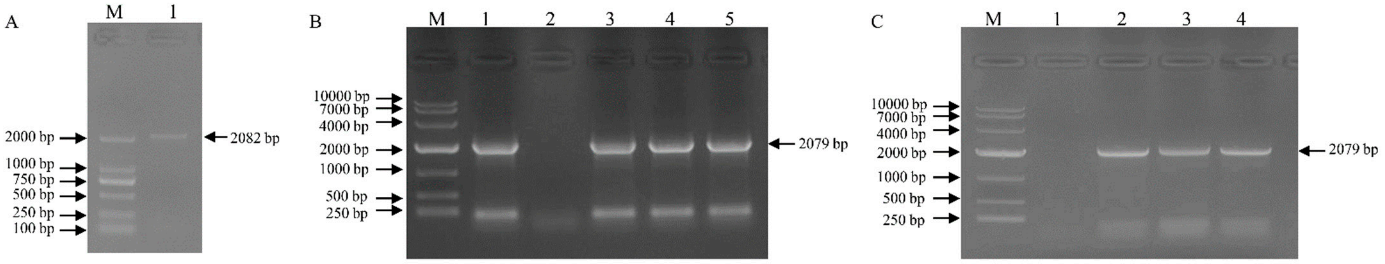

Figure S1. PCR products of *VcSULTR2;1c* and validation of pGDG-VcSULTR2;1C-YFP bacterial solution. A: PCR products of *VcSULTR2;1c*, M: 2000 kb DNA marker, 1: PCR products of *VcSULTR2;1c*. B: Agarose gel electrophoresis of pGDG-VcSULTR2;1c-YFP recombinant expression vector *Escherichia coli*, M: DL 10 kb DNA marker, 1: Positive control, 2: Negative control, 3~5: PCR products. C: Agarose gel electrophoresis of pGDG-VcSULTR2;1c-YFP recombinant expression vector *Agrobacterium tumefaciens*, M: DL 10 kb DNA marker, 1: Negative control, 2~4: PCR products.
