# Supplementary material for: Genome-Wide Identification of the Sulfate Transporters Gene Family in Blueberry (Vaccinium spp.) and Its Response to Ericoid Mycorrhizal Fungi
Source: Int J Mol Sci. 2024 Jun 26;25(13):6980. doi: 10.3390/ijms25136980 (PMC11241426; doi:10.3390/ijms25136980)
Supplement: Supplementary file 1 [file ijms-25-06980-s001.zip › Figure S2.pdf]

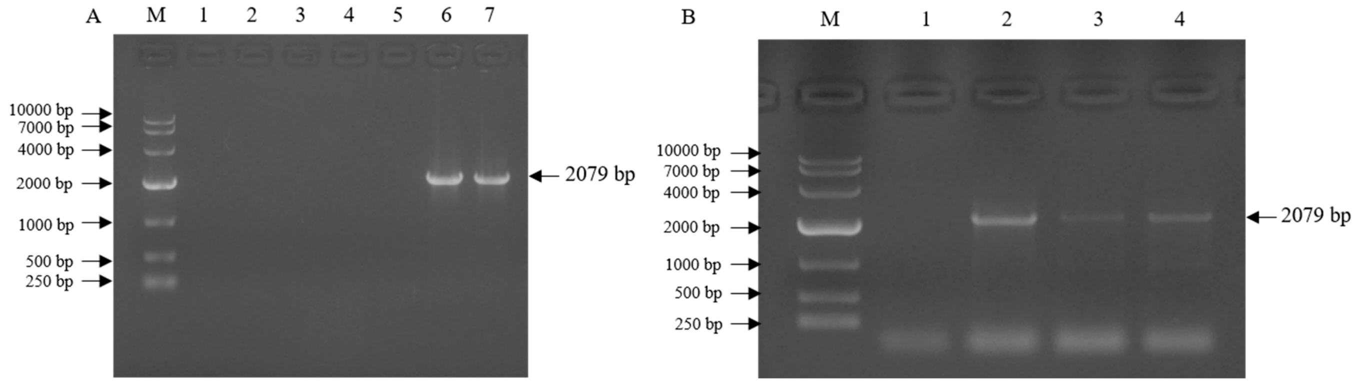

Figure S2 PCR validation of pE2-VcSULTR2;1c bacterial solution. A: Agarose gel electrophoresis of pEASY-VcSULTR2;1c recombinant expression vector *Escherichia coli*, M: DL 10 kb marker, 1: Negative control, 2~7: PCR products; B: Agarose gel electrophoresis of pEASY-VcSULTR2;1c recombinant expression vector *Agrobacterium tumefaciens* in BL21(DE3), M: DL 10 kb marker, 1: Negative control, 2~4: PCR products.
