# Supplementary material for: Genome-Wide Identification of the Sulfate Transporters Gene Family in Blueberry (Vaccinium spp.) and Its Response to Ericoid Mycorrhizal Fungi
Source: Int J Mol Sci. 2024 Jun 26;25(13):6980. doi: 10.3390/ijms25136980 (PMC11241426; doi:10.3390/ijms25136980)
Supplement: Supplementary file 1 [file ijms-25-06980-s001.zip › Figure S3.pdf]

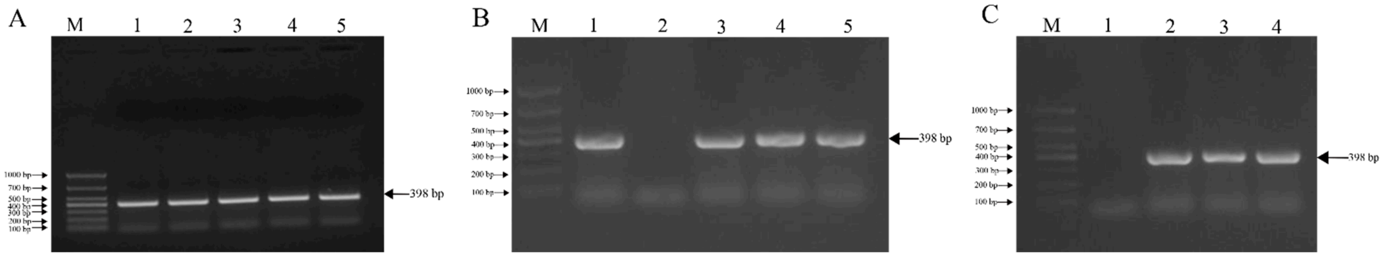

Figure S3 PCR products of pTRV2-VcSULTR2;1c and validation of pTRV2-VcSULTR2;1c bacterial solution. PCR products of pTRV2-VcSULTR2;1c; B: Agarose gel electrophoresis of pTRV2-VcSULTR2;1c, M: 1000 bp Marker, 1: Positive control, 2: Negative control, 3~5: PCR products; C: *Agrobacterium tumefaciens* PCR of pTRV2-VcSULTR2;1c, M: 1000 bp Marker, 1: Negative control, 2~4: PCR products.
