# Supplementary figures and images for: Genome-Wide Identification of the Sulfate Transporters Gene Family in Blueberry (Vaccinium spp.) and Its Response to Ericoid Mycorrhizal Fungi
Source: Int J Mol Sci. 2024 Jun 26;25(13):6980. doi: 10.3390/ijms25136980 (PMC11241426; doi:10.3390/ijms25136980)

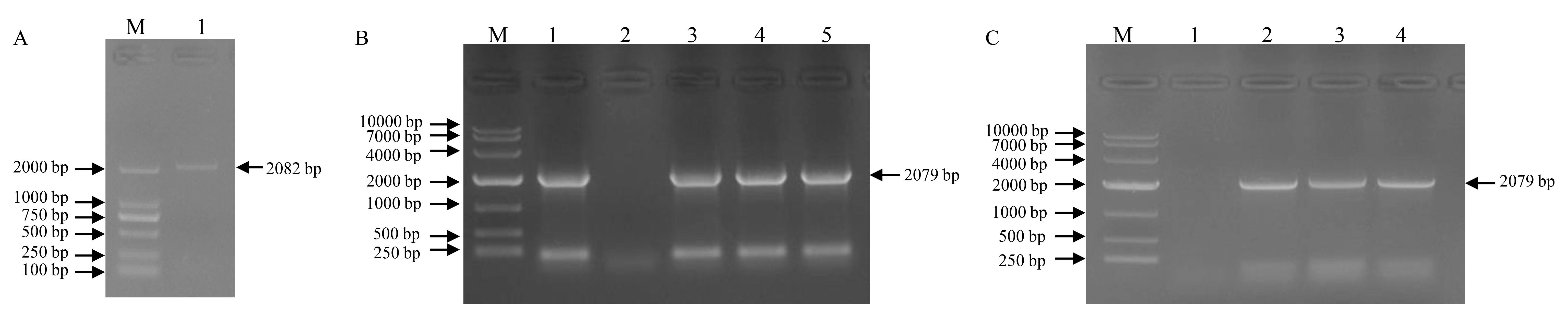

Supplement: Supplementary file 1 [file ijms-25-06980-s001.zip › Figure S1.png]

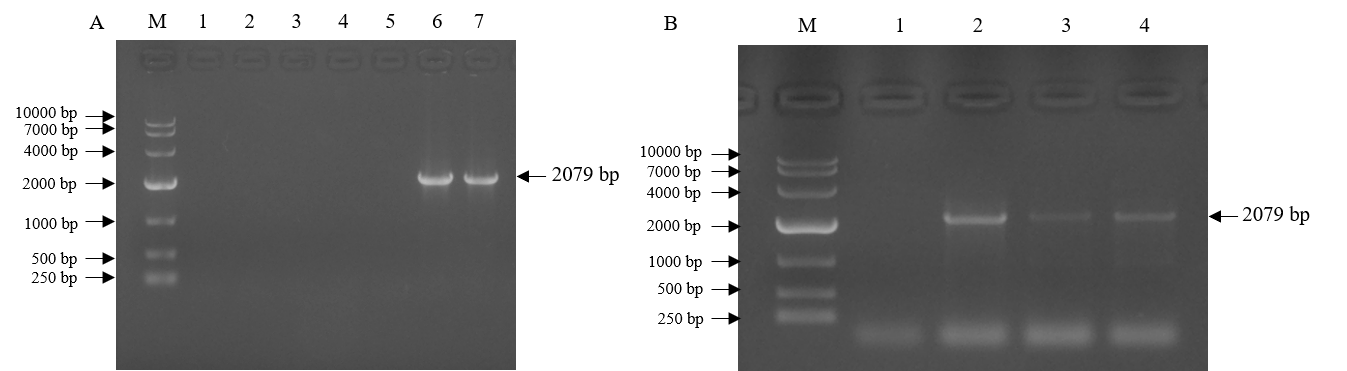

Supplement: Supplementary file 1 [file ijms-25-06980-s001.zip › Figure S2.png]

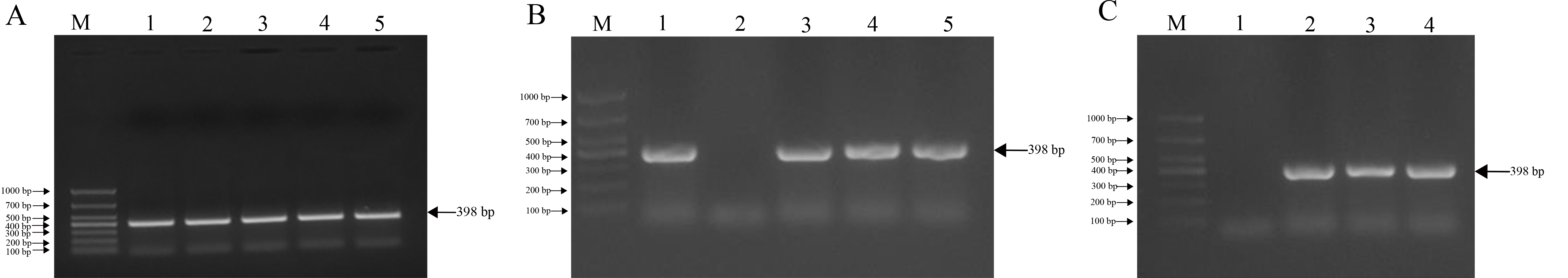

Supplement: Supplementary file 1 [file ijms-25-06980-s001.zip › Figure S3.png]
